# Crystallinity and Liquid Crystallinity of Polyurethanes: How Tailoring of Order Contributes to Customized Properties and Applications

**DOI:** 10.3390/polym17060784

**Published:** 2025-03-15

**Authors:** Artur Bukowczan, Konstantinos N. Raftopoulos, Krzysztof Pielichowski

**Affiliations:** Faculty of Chemical Engineering and Technology, Cracow University of Technology, Warszawska 24, 31-155 Kraków, Poland; konstantinos.raftopoulos@pk.edu.pl

**Keywords:** polyurethanes, crystallinity, liquid crystals, mesogens, self-assembly, order in polymers, shape-memory effect

## Abstract

Studies on polyurethane (PU) materials offer advantageous properties utilized in various applications. The complex nature of the PUs structure and morphology gives them unique properties, but at the same time poses a considerable challenge for the characterization and design of structure–property relationships. Polyurethanes with tailored crystallinity can exhibit peculiar resistance to mechanical and chemical factors, allowing a widening range of application. Liquid crystalline polyurethanes have gained renewed interest thanks to the development of research methodologies and new possibilities for modifying diol and isocyanate monomers. The study shows that liquid crystal phenomena in polyurethanes can be effectively used for polymer compatibilization, in the fiber and nanofibers applications, as well as in ‘smart’ multi-stimuli materials.

## 1. Introduction

One of the reasons for the extensive use of polymeric materials in most applications is the versatility of properties that they can provide depending on their chemical structure. The tuning of these properties, or even the emergence of new ones, can be achieved if two or more types of polymers are incorporated in the same structure. Among the various chemical approaches, polyaddition reactions between monomers or oligomers with properly chosen chemical functionalities are frequently used. A convenient and widely applied reaction in this method is between hydroxyls and isocyanate groups, resulting in the connection of two chain fragments with a urethane link. The resulting polymers—polyurethanes—are a class of versatile polymers; depending on the choice of the reagents, the composition, the technology of production, and different PU materials with tunable properties can be obtained. The chemistry and technology of these materials is well known, and this knowledge is summarized in several excellent books [1,2,3,4,5]. However, a less coherent description on how tailoring of order contributes to desired properties and applications is provided in the literature. Hence, in the current short review, we focus on the types of orders that can occur in polyurethanes and how this ordering can be used in various applications. Special attention will be paid to liquid crystallinity of polyurethanes—an issue that still poses some questions.

**Figure 1:**
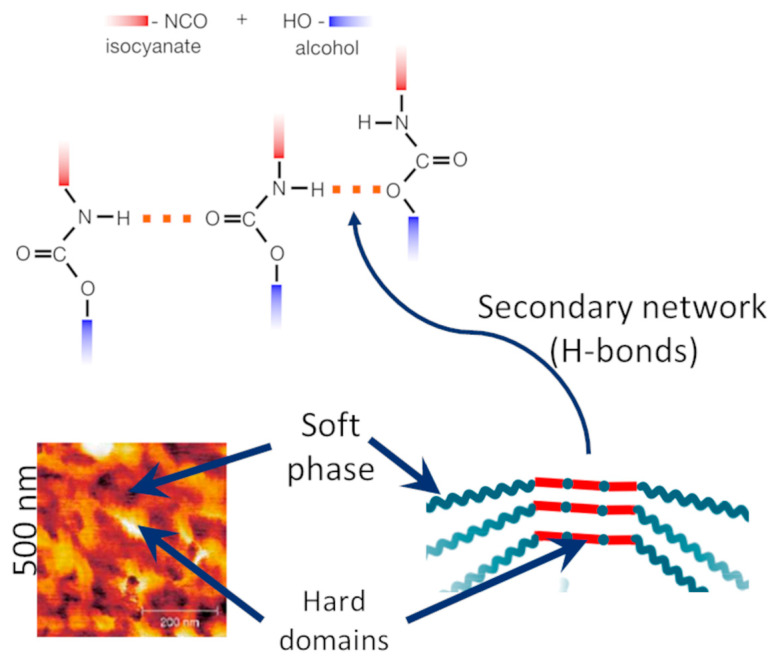
Order formation in standard polyurethane elastomers.

In order to understand how order formation proceeds in polyurethanes, we present some key remarks regarding PU macromolecular structure. In the simplest case (Figure 1), which we will name in the following ‘standard polyurethane elastomers’, the polyurethane is linear, consisting of two types of segments: The first one, called ‘soft segments’, originates from a flexible macrodiol, usually a polyether or polyester. The second, known as ‘hard segments’, is an alternating sequence of fragments originating from a short diisocyanate and a short diol (also known as the chain extender), interconnected with urethane linkages. The hard segments thus produced are obviously rich in urethane groups distributed along the chain in a strictly periodic manner. In addition, urethane groups contain both hydrogen donors (amine) and acceptors (carbonyl group of the carbamide). As intuitively expected, a secondary network of hydrogen bonds is formed (Figure 1), which separates the hard segments from the soft ones. Mesoscopically, this is translated as a phase separation of hard domains consisting almost exclusively of hard segments, distributed in a continuous soft phase consisting mostly of soft segments.

Each of the two phases mentioned above may have an internal order at the molecular level. Most notably, within the hard domains, the usually periodic segments may form well-ordered structures held together by hydrogen bonds between the carbonyl group of the carbamide acting as the proton acceptor and the amine group acting as the proton donor. These structures are periodic at the molecular level and can be considered as crystalline, even though hydrogen bonding occurs at points relatively distant from each other. The study of morphology requires sophisticated time- and temperature-resolved x-ray experiments, which are the decisive method to confirm whether hard domains are crystal-line. The mechanism of ordering, whether it is simply phase separation or accompanied by atomic-level crystallization, has already been extensively studied since the 1980s in the seminal works of Koberstein [6,7,8,9,10,11]. Nevertheless, due to its extreme complexity, interest, and experimental challenges, it has attracted attention up to the early 2020s, with some works pointing out that multiple mechanisms may occur during this ordering [12,13,14,15]. The ongoing interest in this old question is also confirmed by the existence of recent review articles on microphase separation and its experimental study [16]. With the increasing abundance of computational resources, researchers also return to the question of modelling of the undoubtedly complicated processes [17].

**Figure 2:**
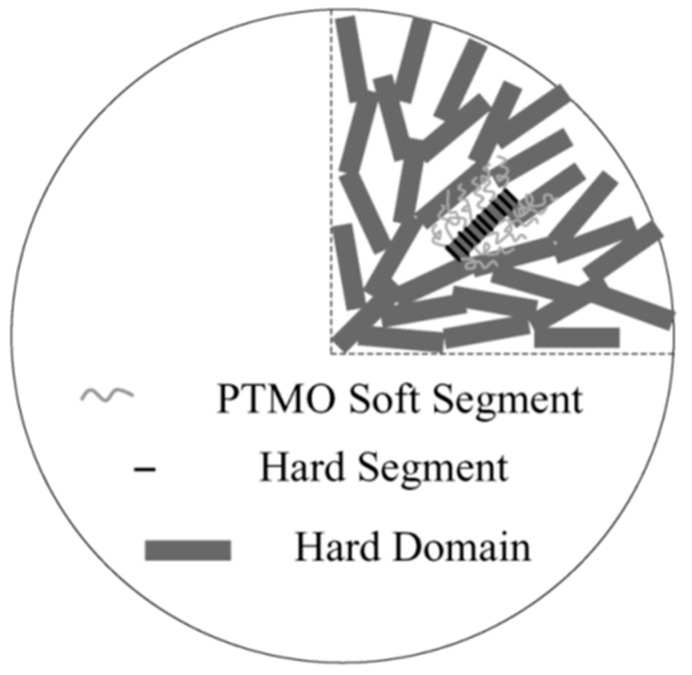
Various arrangements of hard segments in a polyurethane. Reprinted from [19] with permission from Elsevier.

In any case, hard domains have been shown to form larger scale structures of all kinds, including dendrites and lamellae in a fashion very similar to that of all crystalline polymers [18,19]—Figure 2.

A detailed account of the mechanisms of ordering in PUs is well beyond this applications-focused short review; however, we would like to point out that the consensus seems to be that

1. The longer the hard segments, the more likely that they will associate with each other and form ordered structures [18].
2. The larger the mass fraction of hard segments, the more complete the phase separation [20].
3. The final morphology is extremely sensitive to the thermal history of the material with three distinct temperature regions where intrinsic nucleation competes with extrinsic one [8].
4. The kinetics of nucleation and growth depends also on the nature of the soft segment [21].
5. Crosslinking in the soft phase may reduce the ability of phase separation of the hard domains [22,23,24].
6. The chemical nature and the flexibility of the isocyanates also play a role in more flexible aliphatic ones, leading to more ordered structures [15,25]
7. Nanoparticle additives may have an impact on the rate of both the nucleation and growth stages. This impact may be enhancing [26] or hindering, depending not only on the combination of polymer and nanoparticle but also on the temperature of annealing and the mechanism of nucleation [14,27].

Turning our attention to the soft phase, molecular order forms within this one too, provided that the flexible macrodiol itself is crystalline. An additional condition is that it should be sufficiently long and have a high enough mass ratio so that interchain association is possible; crosslinking hinders crystallization ability, as is the case for homopolymers. A higher degree of phase separation also promotes ordering, as diluted hard segments can act as impurities [21,28,29,30]. The degree of crystallinity of the soft phase has a large impact on Young’s modulus of polyurethanes [17,31].

So far, we can see that ordering in conventional polyurethanes occurs in two stages, one with phase separation and one more in internal order within each phase. This second one depends on the respective properties of each phase, taking into account the confinement within the other phase and the mutual interactions. The question then arises whether it is possible to synthesize polyurethanes incorporating segments which show other types of ordering, in a way that this ordering can be retained within the elastomeric matrix. This is of practical importance with respect to the production of materials with the ordered properties given combined with good mechanical behavior, which can be provided by the incorporation within the PU matrix. Among those potential segments, liquid crystalline mesogens are probably the most interesting from both the fundamental and applications point of view [29,32,33]. These compounds in the pure state show several types of long-range ordering depending on the temperature (called thermotropic) or their exposure to a solvent (called lyotropic). They may show orientational long range order, without positional one, in which case the morphology is called ‘nematic’, or they may simultaneously show positional and orientational order, named ‘smectic’ morphology. However, the variations at the actual morphology in the molecular level are quite complicated and found in many variants. Mesogenic units can be incorporated within the polyurethane structure either as diisocyanates or as diols. The interesting issue here is that the ‘liquid crystalline’ ordering may compete with the association of urethane units described above, so its order-forming ability is hindered. A common solution is to introduce flexible spacers between urethane units and mesogen, as well as between urethane units themselves. However, this should be carried out carefully because too low a concentration of mesogenic units can lead to the complete disappearance of the liquid crystalline order. Although final morphology is of great interest with respect to the macroscopic properties of the materials, we are not aware of any systematic fundamental studies. This may arise from the high degree of complexity of those systems, which makes the drawing of general guidelines on structure–properties relationships rather difficult. After all, it should be kept in mind that even the question of ordering of simple polyurethanes is still being investigated after almost a century since their introduction by Otto Bayer. So remains the question of ordering in liquid crystals. The combination of both is, understandably, a very difficult endeavor, which, in addition, requires sophisticated chemistry as well as advanced morphological study, e.g., with temperature-resolved scattering methods. Therefore, in the literature, as we will show, each case is treated individually with respect to a pre-defined application for each time.

In this focused review, we will summarize the impact of morphology on applications, hoping to provide some guidelines for the future development of polyurethanes with tailored ordering. We will start by demonstrating the impact of crystallinity of standard polyurethanes in novel applications. Then, we will continue with a more extensive description of the impact of liquid crystallinity on specific properties of mesogen-bearing polyurethanes.

## 2. The Use of Polyurethane Crystallinity in Targeted Applications

Obtaining polyurethanes with targeted properties by modifying their degree of crystallinity is a multi-level issue. Typically, research focuses on obtaining specific functional properties, where the assessment of the degree of crystallinity is an element that explains the behavior of the material and not its purpose, in contrast to liquid crystalline polyurethanes, where it is crucial to obtain stable mesophases, which consequently allows the material to be used in a specific environment. Nevertheless, there are works that link, to a greater or lesser extent, modifications of the crystallinity of polyurethanes with their functional properties [34,35]. In most cases, we will discuss the influence of crystallinity on mechanical properties, the appropriate level of which determines the use of the materials in the dedicated application field.

In the study of Hossieny et al. [36] authors indicated a direct influence of the distribution of rigid domains on the nucleation properties of polyurethane foams. Consequently, by modifying the crystalline properties, it is possible to influence the functional properties of materials. Indirect tailoring of mechanical properties by changing the degree of crystallinity of polyurethanes was also studied. The presence of a specific aromatic structure in the isocyanate component can induce the formation of crystalline microstructures, leading to a higher mechanical response of the material [37]. On the contrary, the presence of methylene side groups in the diol component can disrupt microphase separation and limit the applicability of the studied material [17]. Adjusting the mechanical properties of polyurethanes by maintaining the proper crystalline level may determine the possibility of using the material as a biocomponent. In the work [38], the authors propose block polyurethanes with controlled structure with significant hemocompatibility where a suitable crystallinity level leads to desirable mechanical response. The direct implication of the soft segment crystallization level on the application of polyurethanes in water-resistant materials was presented in [39]. Using poly (1,4-butylene adipate) with different molar masses, the authors were able to obtain highly crystalline waterborne polyurethanes, which resulted in better water-resistant properties, thus expanding the range of PU-based materials applications. Another study demonstrating the importance of the crystalline level of materials based on PU in practical use was presented by Wu et al. [40]. The incorporation of the crystalline phase improved the mechanical properties of the self-healing PU-based material. Eliminating the basic disadvantage of poor resistance to mechanical forces allowed for reconsideration of the use of polyurethane in medical applications. Inducing crystallinity was also found to be beneficial in PU systems like shape memory materials [41,42,43,44], sensors [45], adhesives [46], and tissue engineering scaffolds [47].

## 3. Liquid Crystalline Polyurethanes (LCPU)

### 3.1. New Generation of Liquid Crystalline Materials and Their Composites

**Figure 3:**
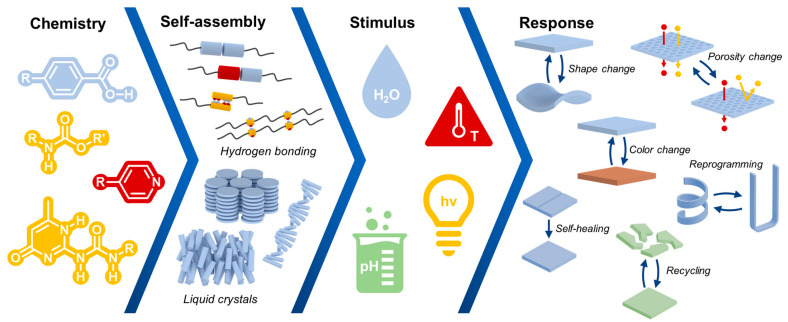
Application of LC materials. Reprinted from [51] with permission from RSC.

Research on liquid crystallinity (LC) phenomena in polymeric materials has been ongoing for several decades [48,49,50]. It is, therefore, no wonder that scientists are looking for modern solutions to apply LC properties in areas such as biomaterials, medicine, electronics, or constructions. The most prominent application of molecular order of LC systems was found to be multi-stimuli devices with tunable properties [51] (Figure 3). There are a number of variables that create systems that often interact with each other, from materials sensitive to pH or water to thermally and radially induced structures. This group of materials is a specific response to meet high demands, particularly in the field of biomedical materials used as wound dressings, artificial muscles and tissues, indicators, and membranes [52,53].

Biomedical applications are not the only key focus of contemporary research on liquid crystalline materials; the reinforcement of engineering materials has also been widely studied, with the reinforcement of polymeric matrices [54,55], 3D printed materials with advantageous mechanical properties [56,57], and fire-resistant systems [58] being the most researched amongst them.

LC materials with self-assembly properties are also used as an effective matrix for nanomaterials [59]. A vast number of works show the beneficial effect of combining LC phenomena with nanomaterials such as metals and metal oxides [60,61,62], graphene [63,64], carbon nanotubes [65,66], or polyhedral oligomeric silsesquioxanes (POSS) [67,68,69].

Before we move on to the analysis of specific works, it is also worth noting that, in the case of liquid crystalline polyurethanes, the connection between crystallinity and applications is more direct. In this case, the introduction of an element with mesomorphic properties is crucial and directly affects the application properties. The range and type of occurrence of liquid crystalline phases are important, to a lesser extent.

### 3.2. LCPUs and Their Composites—Rediscovered Materials

The first polyurethanes with liquid crystalline properties were reported in early 1980 by Iimura et al. [70]. In the following years, several works were published on the use of various components and their impact on the properties of liquid crystals [33]. Despite such extensive research in this field, in the early 2000s, there was no clear indication of the applications of LCPU. One can hypothesize that the majority of the LCPU materials described do not demonstrate sufficient processing properties that justify their use on a larger scale. Together with new methods of synthesis and characterization of polymeric materials, especially in the field of obtaining new monomers in polyurethane technology, the phenomena of using liquid crystalline was rediscovered.

One of the first works on LCPU displaying features other than those described in the literature so far was presented by Wang et al. [71]. In their study, a mesogenic unit with different spacer lengths was used to form side chain LCPU elastomers. The obtained materials exhibited a smectic liquid crystalline phase with mechanical properties similar to those of a regular polyurethane. Following the concept of preparing LCPU elastomers [72], the authors used 4-x-hydroxyhexyloxycarbonyl-4-hexyloxyphenylbenzoate (M6) as a mesogenic pendant group, directly attached to the chain extender. Non-segmented side chain liquid crystalline polyurethanes showed an increased thermal stability compared to the regular polyurethane, additionally exhibiting mesomorphic properties. Another interesting solution was the introduction of liquid crystal cholesteric molecules into the polyurethane chain [73].This LCPU system exhibited a wide cholesteric phase transition with a spherulite crystal structure strongly dependent on the load of the mesogenic unit. A similar composition is presented in the work of the Balenko and co-workers [74]. However, in this case, the cholesteric particles constitute a separate phase dispersed in the polyurethane matrix and show an effective selective light reflection band (SRB). It was stated that these systems can be applied as sensors with reversible color change or other photonic materials. An LCPU system with cellulose that is non-covalently bonded to a polyurethane matrix was described by Han et al. [75]. Although the materials presented showed very good cytotoxic properties, it should be noted that the crystallinity of the matrix was also disturbed, and the nonhomogeneous LC domains were formed. Therefore, it could be concluded that more stable systems combine matrices with LC moieties that are covalently bonded.

An interesting approach to modify the LPCU to improve its processability is described in [76]. By forming a thio-urethane linkage supported by hydrogen bonds and surrounded by appropriate flexible segments, the authors managed to prepare materials that could be processed with various techniques. Moreover, the obtained LCPU systems exhibited a wide range of liquid–crystalline phase transitions and high thermal stability. Several other works [77,78,79,80] showed the beneficial effects of introducing liquid crystal properties into polyurethanes, also in the field of biodegradable materials [81].

### 3.3. Modifications of Materials by LCPU Addition

**Figure 4:**
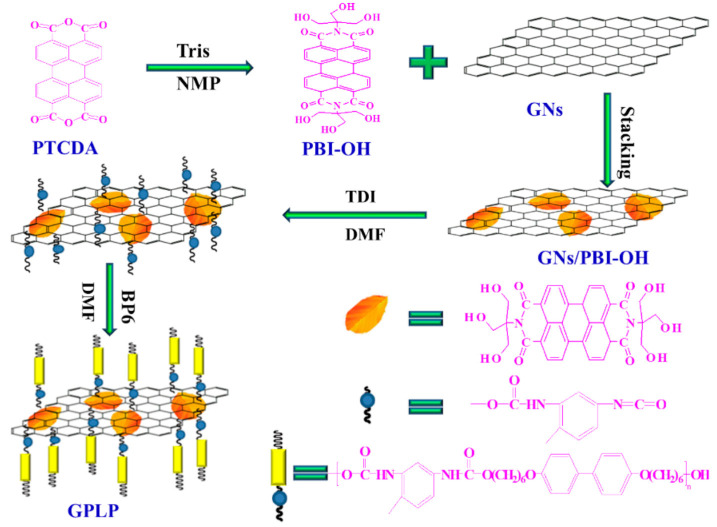
LCPU/GP preparation scheme. Reprinted from [85] with permission from MDPI.

The modification of epoxy resins with liquid crystalline polyurethane-imide was the aim of the study by the Gui group [82]. Significant improvement in the thermal and mechanical properties of the LCPU/epoxy blends was noted. Additionally, a wide range of liquid–crystalline phase transition temperatures was observed, thus increasing the applicability of the systems. The good miscibility and self-assembly of LCPU was found to be an interesting solution to compatibilization issues between epoxy resins and reduced graphene oxide (GO). In the work presented by Zeng et al. [83] authors used a specific structure of perylenetetracarboxylic derivatives to combine graphene oxides with LCPU, then use it as a filler in the epoxy resin matrix. It was found that even a small amount of the LCPU/GO component results in a significant increase in the mechanical and thermal properties of epoxy resins. A similar effect was observed for grafted GO on LCPU [84]. The compatibilization effect of LCPU was also studied for graphene nanosheets and epoxy resins [85]. Here, authors used perylene derivatives in order to form π-π stacking interactions with LCPU, and the obtained hybrids were used as filler for epoxy resins—Figure 4. As in the composites mentioned above, the hybrid-supported systems exhibited higher tensile strength, Young’s modulus, toughness, and enhanced thermal stability.

With an increasing interest in new liquid crystalline materials, attempts have also been made to combine mesomorphic properties with macro- and nano-additives. Lu et al. [86] presented composites based on epoxy resin filled with nano Al_2_O_3_, where LCPU was used as a toughening modifier. Interestingly, the addition of LCPU itself improved the mechanical and thermal properties. The effect of nano Al_2_O_3_ was largely dependent on its amount and the degree of homogenization in the polymer matrix.

The effect of metallic nanoparticles on the LCPU matrix was also studied [87]. ZnO and TiO_2_ nanoparticles were first grafted with organic compounds in order to improve compatibility and then added to the LCPU matrix. Although the study is only a preliminary one, authors conclude that LC phase transitions were not disturbed even when the morphology was changed.

Another study of LCPU nanocomposites focused on the addition of POSS nanoparticles [67,69]. Nanofiller was added as the reactive component bearing three hydroxyl groups capable of reacting with isocyanate during formation of LCPU chains. Such modification led to the widening of the liquid crystalline phase, together with a higher mechanical response to stretching and increased thermal stability. Moreover, the influence of the different architectures of POSS moieties on the charge flow and the thermal degradation path was also studied [68,88].

The systems and their properties described above were used to prepare a new class of materials: multi-stimuli and shape-memory liquid crystalline polyurethanes, and their composites.

### 3.4. “Smart” Liquid Crystalline Polyurethane Composites

In recent years, LCPUs have been applied in the field of multi-stimuli and shape-memory systems. Combining a variety of different properties to create new, unique, and multifunctional materials has become the main goal of modern materials engineering. Yu and coworkers published one of the first works describing a possible application of LCPU [89]. The authors incorporated azobenzene moieties to form liquid crystalline polyurethane films. The results showed some promising photomechanical properties that indicate the wide possibilities for modification and application of the tested materials. Azobenzene based liquid crystalline polyurethanes were also the focus of Wen et al. [90]. Here researchers prepared a variety of side-chain liquid crystalline polyurethanes with different chain lengths. The smectic C phases were determined with a transition temperature of ca. 40 °C, which triggers a triple-shape-memory effect. By changing the orientation of the LC phase and the cross-linking densities during the tests, an effective shape-memory recovery was enabled. Under UV-Vis irradiation, SCLP (side chain liquid crystalline polymers) exhibited bending–unbending behavior within the range of 450–550 nm. The combination of two types of mesogenic moieties in LCPU: azobenzene and nematic carbonitrile derivative was studied in [91]. By using a quaternization method, a liquid crystalline side chain was introduced into the polyurethane main chain and then a photosensitive unit was grafted on it. The resulting ‘smart’ material exhibited photoisomerization together with self-healing properties from the second mesogenic unit. It was hypothesized that such a solution can extend the service life of the material, which is particularly important in the field of optomechanical devices. Similar research with the use of azobenzene moieties was described in [92,93,94,95]. As an individual mesogenic unit, a carbonitrile derivative that forms LCPU was studied by Wen et al. [96]. In their work, the authors proposed a system with variable molar masses and cross-linking densities, which allowed for the investigation of the most effective amount of cross-linking agent. Triple-shape-memory properties were studied and the relationship between the chain structure and the range of effective phase transformation has been proposed.

Among the mesogens used in the ‘smart’ LCPUs, there are also derivatives of benzoic acid, which are characterized by the formation of a reversible nematic phase. The addition of 4-hexadecyloxybenzoic acid (HOBA) to the shape-memory polyurethane system was studied by Chen and co-workers [97]. The prepared composites demonstrated a shift from one-step shape-memory recovery to two-steps after doping the matrix with LC mesogenic unit. However, it is worth noting that the systems described involve a physically mixed polymer–mesogen system, which may consequently lead to the formation of nonhomogeneous regions visible in microscopic examination. The same team also conducted research using the 4-n-octyldecyloxybenzoic acid mesogenic unit bonded by hydrogen bonds with the segmented polyurethanes [98]. The obtained materials maintained liquid crystalline properties of mesogen together with triple- and quadrupole-shape-memory recovery features. Similar systems have also been studied in references [99,100,101,102].

### 3.5. Liquid Crystalline Polyurethane Fibers and Nanofibers

**Figure 5:**
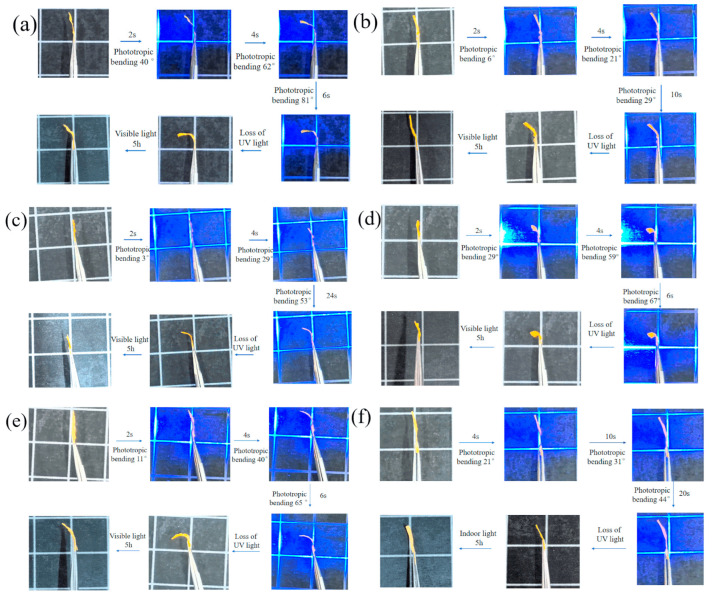
Photoresponsive properties of fibrous LCPU based membranes. (**a**) LCPU-1:SMPU = 1:2, (**b**) LCPU-1: SMPU = 3:4, (**c**) LCPU-1:SMPU = 1:1, (**d**) LCPU-2: SMPU = 1:2, (**e**) LCPU-3: SMPU = 1:2, (**f**) LCPU-3: SMPU = 3:4. Reprinted from [113] with permission from MDPI.

Polyurethane materials play an important role in the polymeric fiber industry [103]. Currently, research on the use of polyurethane fibers focuses, in addition to the field of textiles and furniture [104,105,106,107], from water purification processes [108] to advanced biomedical materials [109,110]. The unquestionable success achieved by liquid crystalline aramid and polyester fibers in the use of high-strength textile materials meant that polyurethane fibers were also modified to acquire mesomorphic properties. Electrospinning of high-performance polymers has an important limitation due to their resistance to heat and solvents. Therefore, the use of this technique for LCPU requires specific conditions and solvents. Bertocchi et al. presented a study of electrospinning a PU blended with the commercial liquid crystalline polymer, Vectran™ [111]. The authors added pentafluorophenol to dissolve LCP in chloroform and then mixed it with the polyurethane solution. This protocol enabled the study of the electrospinning process and the influence of different variables on the formation and alignment of nanofibers. In work [112], the authors used hexafluoroisobutanol as an effective solution for liquid crystalline polyurethane/POSS composites. The study demonstrated the potential of electrospinning LCPU and investigated the influence of different architectures of POSS molecules on nanofiber formation. Advanced solutions for LCPU nanofibers’ formation and application were presented by Zhou and co-workers [113]. Liquid crystalline poly (urethane-acrylates) were blended with shape-memory polyurethanes in order to prepare membranes by electrospinning. The resulting material not only exhibited a fibrous structure but also shape-memory and photoinduced properties (Figure 5).

The systems described above concern liquid crystalline systems where mesomorphic phase change is induced by temperature. This is a typical system for LC polyurethanes and polyesters. However, there are some studies on lyotropic liquid crystalline polyurethanes and the attempt to use them in electrospinning [114]. As for lyotropic materials, the induction of liquid crystalline phase change was performed by choosing a proper solvent and concentration; the authors used a variety of polymer/solvent systems. The possibility of preserving liquid crystalline phases during electrospinning was investigated. As expected, the presence of crystalline regions in a material with a large surface area was found to enhance the antimicrobial activity.

An independent but related example is the use of PU fibers as reinforcement of liquid crystalline nanocomposites [115]. Polysiloxane LCPs exhibiting nematic LC nature were doped with carbon nanotubes and then grafted onto a polyurethane fibrous matrix. The system showed anisotropic properties (both symmetric and asymmetric deformation) with high mechanical resistance of the PU fibers and photothermal properties due to the presence of nanoadditives. Another work on PU fibers as matrices was presented by the Morooka group [116]. They studied inorganic nanosheets with mesomorphic properties covalently bonded with PU prepolymer. It was stated that the presence of PC phases eases the exfoliation process and allows for a good dispersion of nanosheets in the polyurethane fibers. All of these resulted in the significantly increased mechanical and thermal properties of the liquid crystalline composite.

## 4. Conclusions

Despite many years of research, polyurethane materials still attract wide interest in many research branches, particularly in terms of their vast application possibilities. The wide range of possibilities for the use of diol and isocyanate components with different structures allows for continuous improvement in the structure and fabrication of polyurethane materials with desired properties. Taking control of the crystallization processes in polyurethanes is also of great importance from an application point of view. However, the control of ordering in the molecular, supramolecular, and mesoscopic level, is a very complex issue, and its understanding requires more effort from the fundamental research point of view. Phenomena such as microphase separation, crystallinity, and self-assembly in polyurethanes have a very complex nature, giving rise to a number of difficulties associated with their characterization and complete understanding. For example, the question of how the constituting components of polyurethane affect the molecular and mesoscopic ordering presents a new challenges as new types of functional components are incorporated in PU-based materials. It is not, for example, easy to predict whether, and most importantly, under which conditions, the functionality of such components will be retained when the said component is constrained in a polyurethane matrix. It should also be understood whether individual properties, morphological or otherwise, will “compete” with the conventional secondary hydrogen bonding of PUs, or whether they will coexist, and how this synergy will influence the final properties of the materials. Notably, this can lead to the development of effective multi-stimuli materials.

A group of polyurethanes of particular interest are the newly rediscovered liquid crystalline polyurethanes. The potential for their use can be found in their unique adaptive properties, thanks to which one can design a wide range of structures that can create permanent or non-permanent physical and chemical connections. This fact, combined with the liquid crystalline properties, enables their use in advanced shape-memory materials, fibers, and nanofibers, and as efficient compatibilizers for various polymeric systems. The development of the field of liquid crystal polyurethanes may provide an alternative to commercially available liquid crystal polyesters and polyamides. Further developments in this promising field are expected, possibly utilizing bio-based monomers for polyurethane synthesis, as well as non-isocyanate polyurethane (NIPU) chemistries.
